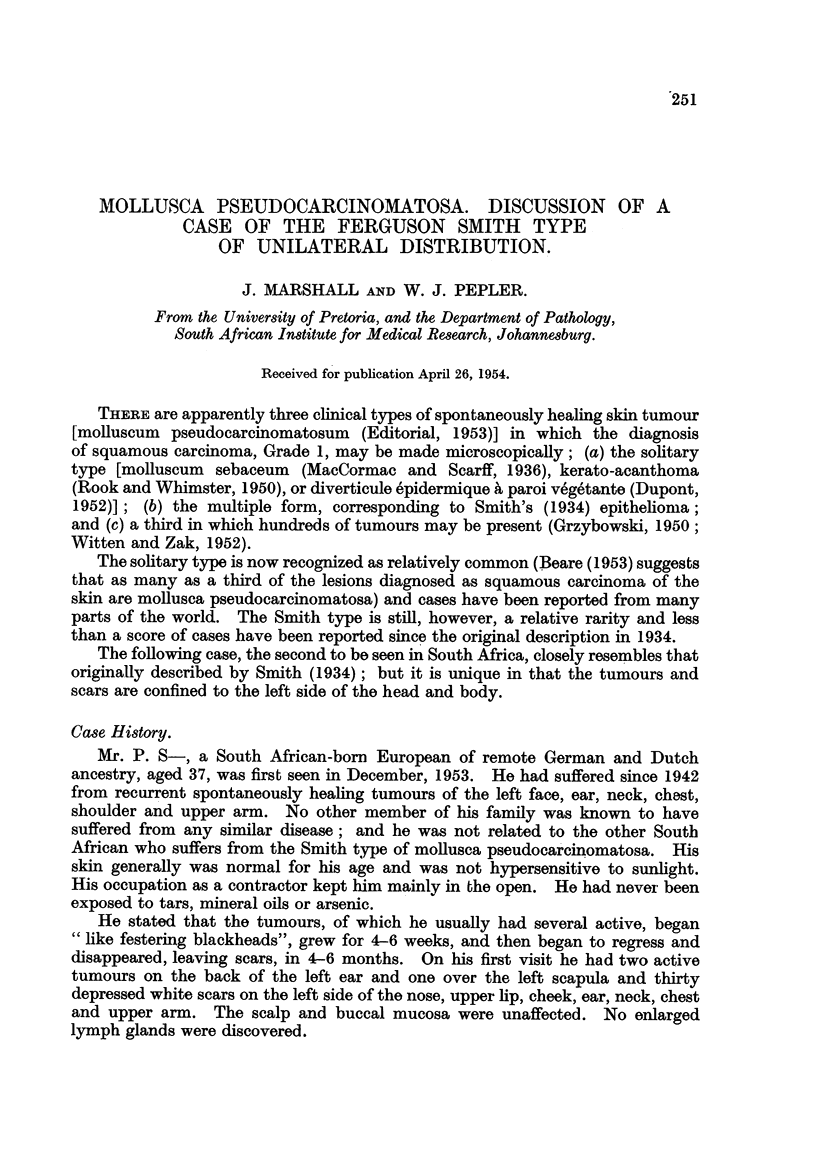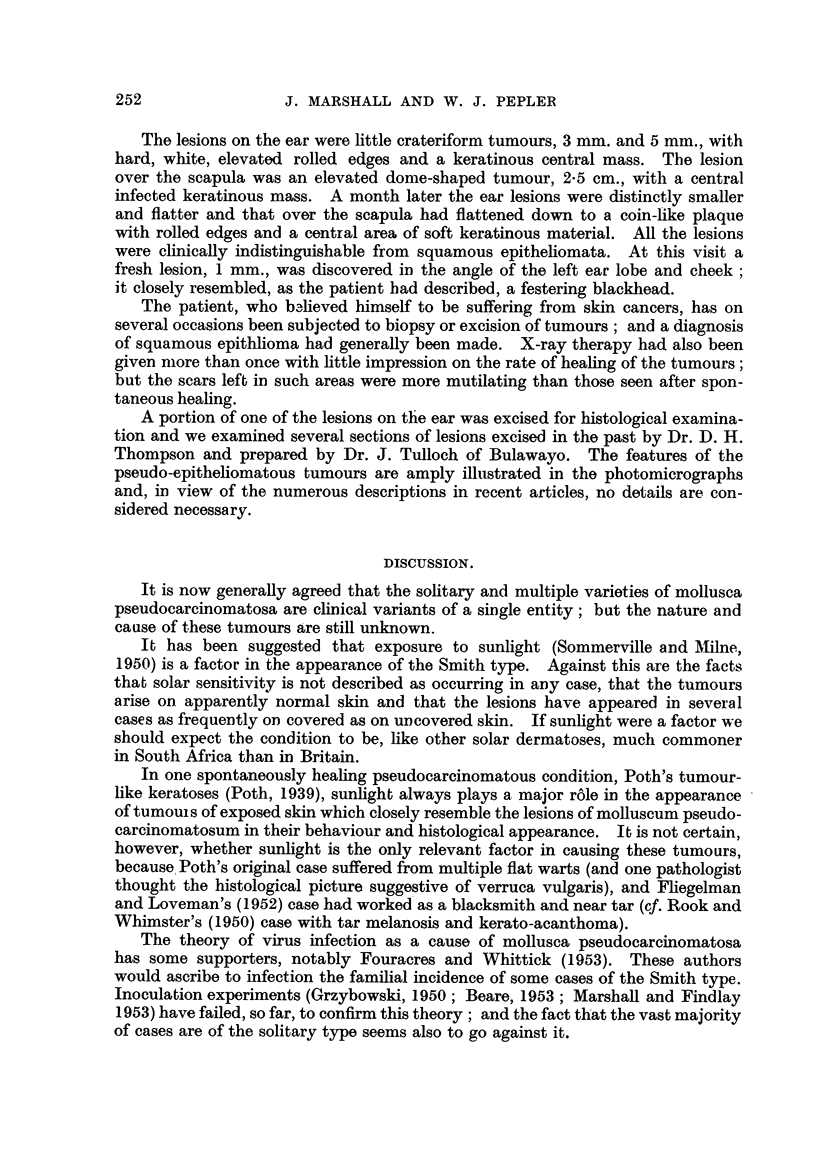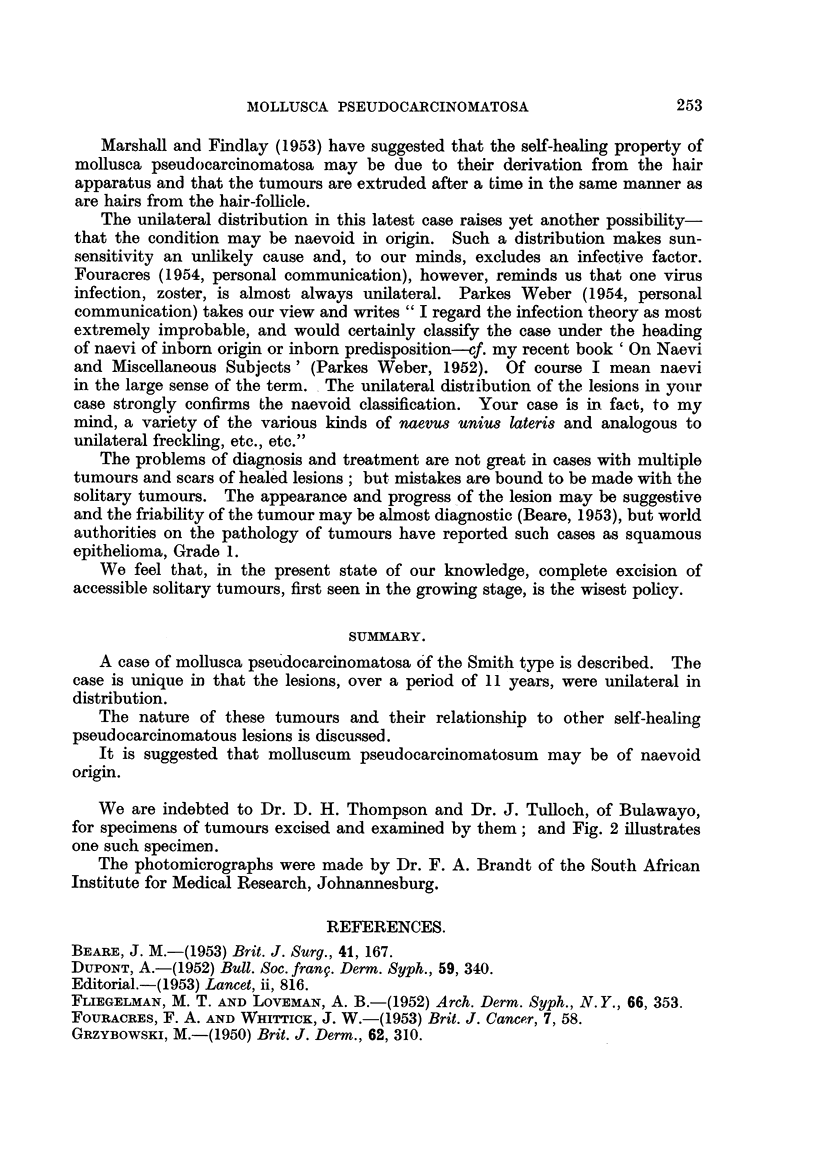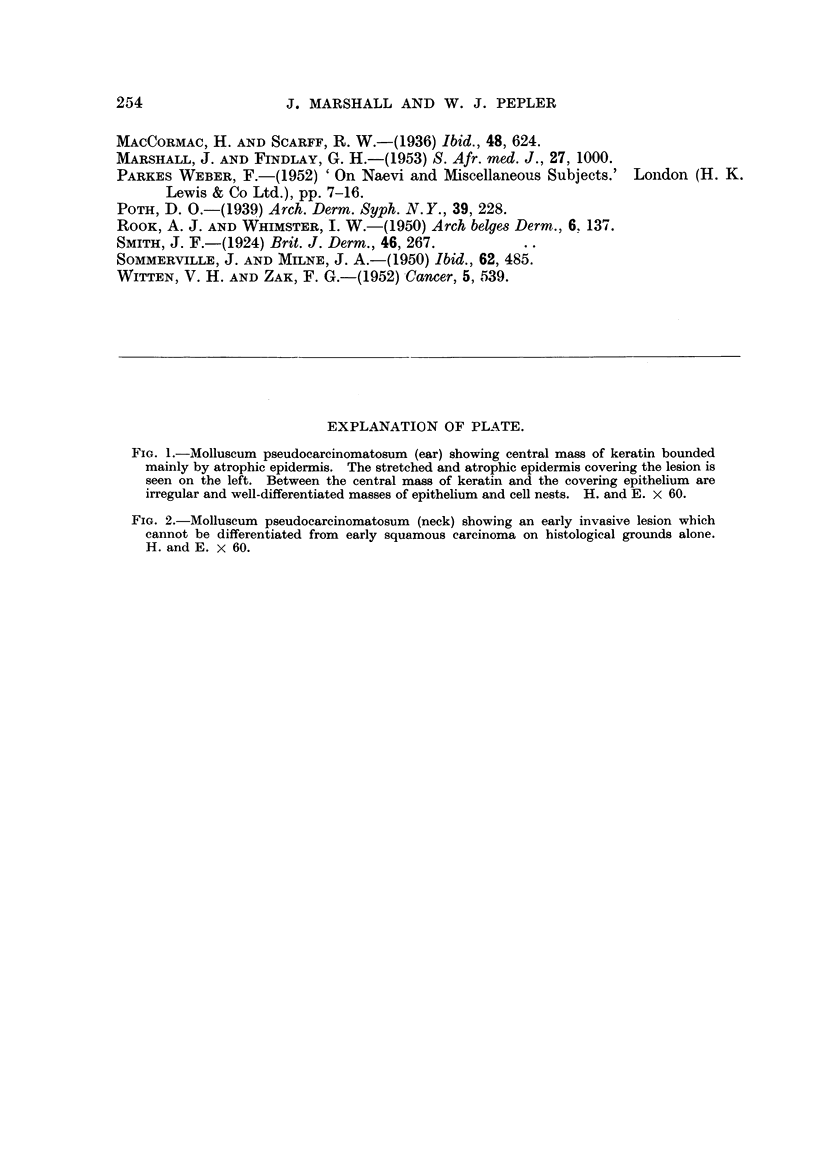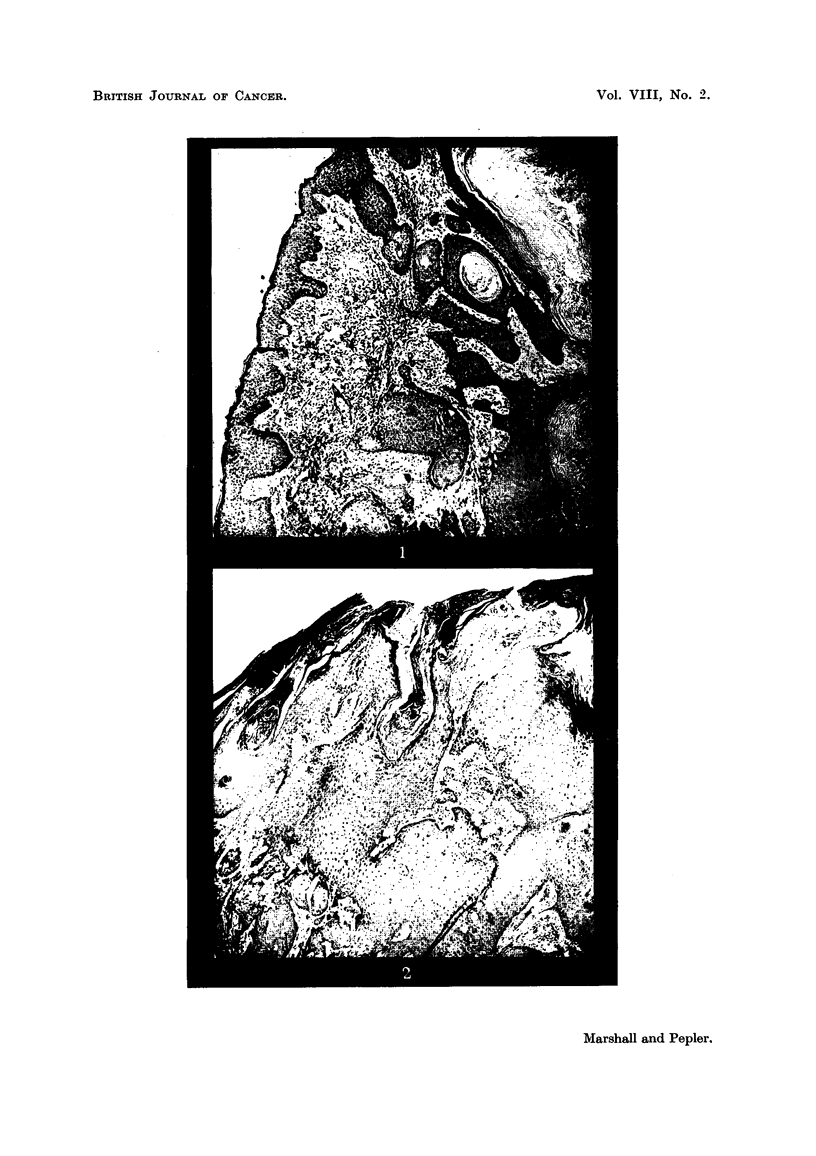# Mollusca Pseudocarcinomatosa. Discussion of a Case of the Ferguson Smith Type of Unilateral Distribution

**DOI:** 10.1038/bjc.1954.25

**Published:** 1954-06

**Authors:** J. Marshall, W. J. Pepler

## Abstract

**Images:**


### MOLLUSCA PSEUDOCARCINOMATOSA. DISCUSSION OF A CASE OF THE FERGUSON SMITH TYPE OF UNILATERAL DISTRIBUTION.

J. MARSHALL AND W. J. PEPLER.

*From the University of Pretoria, and the Department of Pathology, South African Institute for Medical Research, Johannesburg.*

Received for publication April 26, 1954.

THERE are apparently three clinical types of spontaneously healing skin tumour [molluscum pseudocarcinomatosum (Editorial, 1953)] in which the diagnosis of squamous carcinoma, Grade 1, may be made microscopically ; (*a*) the solitary type [molluscum sebaceum (MacCormac and Scarff, 1936), kerato-acanthoma (Rook and Whimster, 1950), or diverticule épidermique à paroi végétante (Dupont, 1952)] ; (*b*) the multiple form, corresponding to Smith's (1934) epithelioma ; and (*c*) a third in which hundreds of tumours may be present (Grzybowski, 1950 ; Witten and Zak, 1952).

The solitary type is now recognized as relatively common (Beare (1953) suggests that as many as a third of the lesions diagnosed as squamous carcinoma of the skin are mollusca pseudocarcinomatosa) and cases have been reported from many parts of the world. The Smith type is still, however, a relative rarity and less than a score of cases have been reported since the original description in 1934.

The following case, the second to be seen in South Africa, closely resembles that originally described by Smith (1934) ; but it is unique in that the tumours and scars are confined to the left side of the head and body.

*Case History.*

Mr. P. S—, a South African-born European of remote German and Dutch ancestry, aged 37, was first seen in December, 1953. He had suffered since 1942 from recurrent spontaneously healing tumours of the left face, ear, neck, chest, shoulder and upper arm. No other member of his family was known to have suffered from any similar disease ; and he was not related to the other South African who suffers from the Smith type of mollusca pseudocarcinomatosa. His skin generally was normal for his age and was not hypersensitive to sunlight. His occupation as a contractor kept him mainly in the open. He had never been exposed to tars, mineral oils or arsenic.

He stated that the tumours, of which he usually had several active, began " like festering blackheads", grew for 4–6 weeks, and then began to regress and disappeared, leaving scars, in 4–6 months. On his first visit he had two active tumours on the back of the left ear and one over the left scapula and thirty depressed white scars on the left side of the nose, upper lip, cheek, ear, neck, chest and upper arm. The scalp and buccal mucosa were unaffected. No enlarged lymph glands were discovered.

The lesions on the ear were little crateriform tumours, 3 mm. and 5 mm., with hard, white, elevated rolled edges and a keratinous central mass. The lesion over the scapula was an elevated dome-shaped tumour, 2·5 cm., with a central infected keratinous mass. A month later the ear lesions were distinctly smaller and flatter and that over the scapula had flattened down to a coin-like plaque with rolled edges and a central area of soft keratinous material. All the lesions were clinically indistinguishable from squamous epitheliomata. At this visit a fresh lesion, 1 mm., was discovered in the angle of the left ear lobe and cheek ; it closely resembled, as the patient had described, a festering blackhead.

The patient, who believed himself to be suffering from skin cancers, has on several occasions been subjected to biopsy or excision of tumours ; and a diagnosis of squamous epithlioma had generally been made. X-ray therapy had also been given more than once with little impression on the rate of healing of the tumours ; but the scars left in such areas were more mutilating than those seen after spontaneous healing.

A portion of one of the lesions on the ear was excised for histological examination and we examined several sections of lesions excised in the past by Dr. D. H. Thompson and prepared by Dr. J. Tulloch of Bulawayo. The features of the pseudo-epitheliomatous tumours are amply illustrated in the photomicrographs and, in view of the numerous descriptions in recent articles, no details are considered necessary.

### DISCUSSION.

It is now generally agreed that the solitary and multiple varieties of mollusca pseudocarcinomatosa are clinical variants of a single entity ; but the nature and cause of these tumours are still unknown.

It has been suggested that exposure to sunlight (Sommerville and Milne, 1950) is a factor in the appearance of the Smith type. Against this are the facts that solar sensitivity is not described as occurring in any case, that the tumours arise on apparently normal skin and that the lesions have appeared in several cases as frequently on covered as on uncovered skin. If sunlight were a factor we should expect the condition to be, like other solar dermatoses, much commoner in South Africa than in Britain.

In one spontaneously healing pseudocarcinomatous condition, Poth's tumour-like keratoses (Poth, 1939), sunlight always plays a major rôle in the appearance of tumours of exposed skin which closely resemble the lesions of molluscum pseudocarcinomatosum in their behaviour and histological appearance. It is not certain, however, whether sunlight is the only relevant factor in causing these tumours, because Poth's original case suffered from multiple flat warts (and one pathologist thought the histological picture suggestive of verruca vulgaris), and Fliegelman and Loveman's (1952) case had worked as a blacksmith and near tar (*cf.* Rook and Whimster's (1950) case with tar melanosis and kerato-acanthoma).

The theory of virus infection as a cause of mollusca pseudocarcinomatosa has some supporters, notably Fouracres and Whittick (1953). These authors would ascribe to infection the familial incidence of some cases of the Smith type. Inoculation experiments (Grzybowski, 1950 ; Beare, 1953 ; Marshall and Findlay 1953) have failed, so far, to confirm this theory ; and the fact that the vast majority of cases are of the solitary type seems also to go against it.

Marshall and Findlay (1953) have suggested that the self-healing property of mollusca pseudocarcinomatosa may be due to their derivation from the hair apparatus and that the tumours are extruded after a time in the same manner as are hairs from the hair-follicle.

The unilateral distribution in this latest case raises yet another possibility—that the condition may be naevoid in origin. Such a distribution makes sun-sensitivity an unlikely cause and, to our minds, excludes an infective factor. Fouracres (1954, personal communication), however, reminds us that one virus infection, zoster, is almost always unilateral. Parkes Weber (1954, personal communication) takes our view and writes " I regard the infection theory as most extremely improbable, and would certainly classify the case under the heading of naevi of inborn origin or inborn predisposition—*cf.* my recent book ' On Naevi and Miscellaneous Subjects ' (Parkes Weber, 1952). Of course I mean naevi in the large sense of the term. The unilateral distribution of the lesions in your case strongly confirms the naevoid classification. Your case is in fact, to my mind, a variety of the various kinds of *naevus unius lateris* and analogous to unilateral freckling, etc., etc."

The problems of diagnosis and treatment are not great in cases with multiple tumours and scars of healed lesions ; but mistakes are bound to be made with the solitary tumours. The appearance and progress of the lesion may be suggestive and the friability of the tumour may be almost diagnostic (Beare, 1953), but world authorities on the pathology of tumours have reported such cases as squamous epithelioma, Grade 1.

We feel that, in the present state of our knowledge, complete excision of accessible solitary tumours, first seen in the growing stage, is the wisest policy.

### SUMMARY.

A case of mollusca pseudocarcinomatosa of the Smith type is described. The case is unique in that the lesions, over a period of 11 years, were unilateral in distribution.

The nature of these tumours and their relationship to other self-healing pseudocarcinomatous lesions is discussed.

It is suggested that molluscum pseudocarcinomatosum may be of naevoid origin.

We are indebted to Dr. D. H. Thompson and Dr. J. Tulloch, of Bulawayo, for specimens of tumours excised and examined by them ; and Fig. 2 illustrates one such specimen.

The photomicrographs were made by Dr. F. A. Brandt of the South African Institute for Medical Research, Johnannesburg.

### REFERENCES.

Beare, J. M.—(1953) *Brit. J. Surg.*, **41**, 167.
Dupont, A.—(1952) *Bull. Soc. franç. Derm. Syph.*, **59**, 340.
Editorial.—(1953) *Lancet*, ii, 816.
Fliegelman, M. T. and Loveman, A. B.—(1952) *Arch. Derm. Syph., N.Y.*, **66**, 353.
Fouracres, F. A. and Whittick, J. W.—(1953) *Brit. J. Cancer*, **7**, 58.
Grzybowski, M.—(1950) *Brit. J. Derm.*, **62**, 310.

MacCormac, H. and Scarff, R. W.—(1936) *Ibid.*, **48**, 624.
Marshall, J. and Findlay, G. H.—(1953) *S. Afr. med. J.*, **27**, 1000.
Parkes Weber, F.—(1952) 'On Naevi and Miscellaneous Subjects.' London (H. K. Lewis & Co Ltd.), pp. 7–16.
Poth, D. O.—(1939) *Arch. Derm. Syph. N.Y.*, **39**, 228.
Rook, A. J. and Whimster, I. W.—(1950) *Arch belges Derm.*, **6**, 137.
Smith, J. F.—(1924) *Brit. J. Derm.*, **46**, 267.
Sommerville, J. and Milne, J. A.—(1950) *Ibid.*, **62**, 485.
Witten, V. H. and Zak, F. G.—(1952) *Cancer*, **5**, 539.

---

### EXPLANATION OF PLATE.

Fig. 1.—Molluscum pseudocarcinomatosum (ear) showing central mass of keratin bounded mainly by atrophic epidermis. The stretched and atrophic epidermis covering the lesion is seen on the left. Between the central mass of keratin and the covering epithelium are irregular and well-differentiated masses of epithelium and cell nests. H. and E. × 60.

Fig. 2.—Molluscum pseudocarcinomatosum (neck) showing an early invasive lesion which cannot be differentiated from early squamous carcinoma on histological grounds alone. H. and E. × 60.